# Vitamin D receptor polymorphisms as predictors of pediatric infectious disease prevalence and outcome: an overview of available evidence

**DOI:** 10.3389/fimmu.2026.1905972

**Published:** 2026-07-28

**Authors:** Maria Oana Săsăran, Claudia Bănescu

**Affiliations:** 1 Department of Pediatrics, George Emil Palade University of Medicine, Pharmacy, Sciences and Technology of Târgu Mureş, Târgu Mureş, Romania; 2 Genetics Department, Center for Advanced Medical and Pharmaceutical Research, George Emil Palade University of Medicine, Pharmacy, Science and Technology of Târgu Mureş, Targu Mures, Romania

**Keywords:** pediatric infections, respiratory tract, urinary tract, VDR polymorphism, vitamin D receptor

## Abstract

**Background and objective:**

Polymorphisms of the vitamin D receptor (VDR) gene have been studied in relation to various diseases, including infections. This narrative review aims to summarize the role of VDR polymorphisms in the susceptibility towards pediatric infectious diseases, as well as in their complications.

**Methods:**

We conducted a narrative review of the available literature data (PubMed/MEDLINE, Web of Science and Google Scholar databases) published until January 31^st^, 2026. Search keywords included “VDR” AND “child” AND “respiratory tract infection” OR “gastroenteritis” OR “diarrhea” OR “urinary tract infection”.

**Results:**

A total number of 21 studies complied to the inclusion and exclusion criteria. *FokI* remains the most intensely studied polymorphism of the VDR gene in relation to pediatric infections and its genotypes are differentially expressed in relation to infections of the respiratory tract requiring hospitalization. There are no studies available analyzing the impact of VDR gene polymorphisms in acute infectious diarrhea, but two studies have shown so far that children who are carriers of particular genotypes of the *ApaI, FokI* and/or *BsmI* polymorphisms are more predisposed towards developing urinary tract infections. Data on pediatric/neonatal sepsis is scarce, limited to three studies which have shown inconsistent, contradictory findings.

**Conclusions:**

Future studies are warranted to validate or infirm the role of VDR gene polymorphisms in the prediction of pediatric infectious disease incidence and outcomes. The heterogeneity of available studies, given by the different age groups, ethnicity of populations enrolled and geographical locations greatly influences available results and their interpretation.

## 1 Introduction

**Figure 1:**
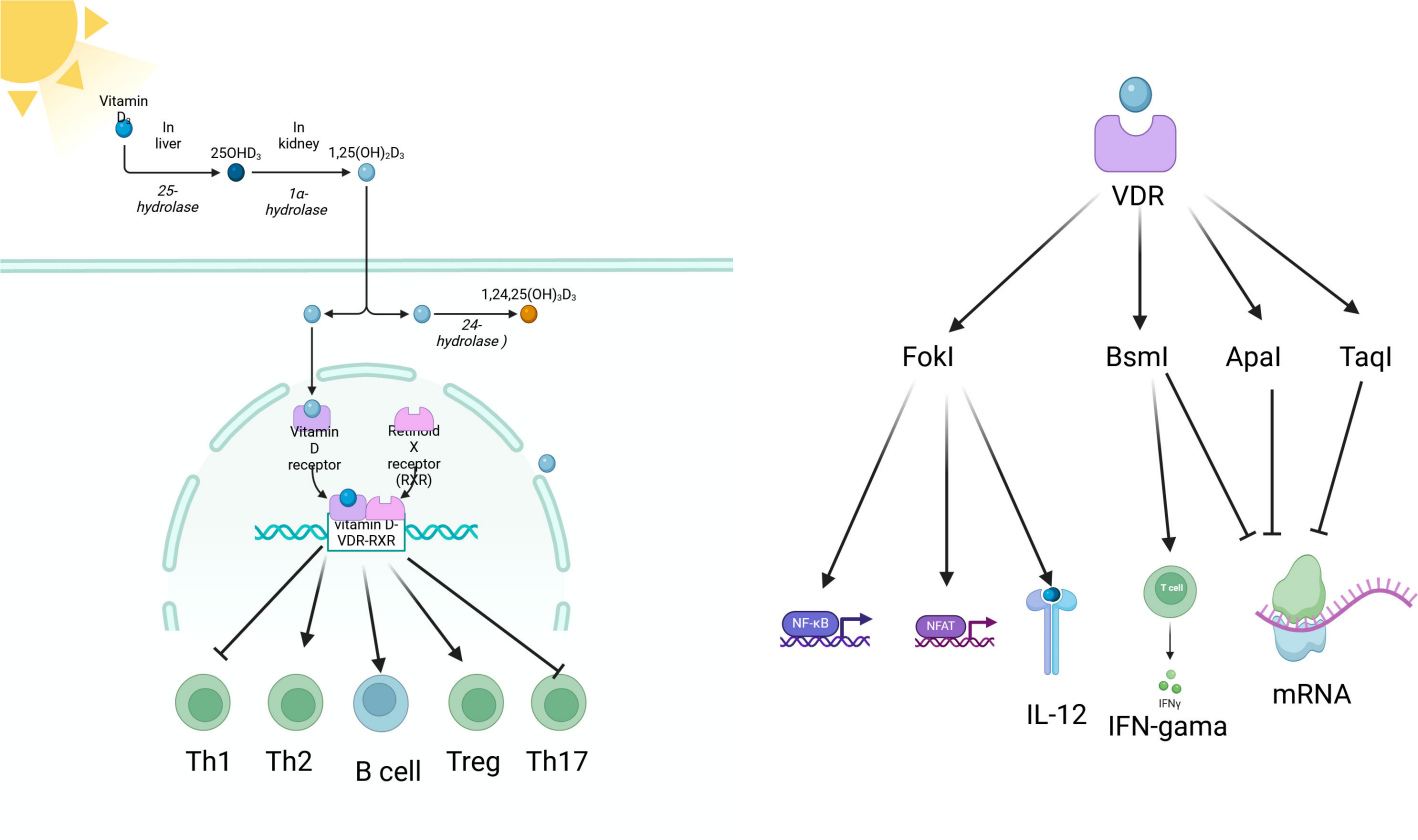
Vitamin D receptor modulation of immune response and function of its common polymorphisms. Created in BioRender. Sasaran, M. O. (2026) https://BioRender.com/asx5bqo. IFN, interferon; IL, interleukin; mRNA, messenger ribonucleic acid; NFAT, Nuclear factor of activated T-cells; Nf-kB, nuclear factor kappa B; RXR, retinoid X receptor; Th, T helper cell; Treg, T regulator cell; VDR, vitamin D receptor.

Vitamin D plays an essential role in bone homeostasis, but also plays a pivotal function in immune regulation and modulation of inflammatory pathways (1). Its effects on immunity are mainly exerted through the nuclear vitamin D receptor (VDR), which can regulate the activity of inflammatory cells, including cytokine production (1, 2). The nuclear VDR represents a protein made up of 437 amino acids and is encoded by the VDR gene, which is situated on chromosome 12 (3, 4). The vitamin D binds to VDR, which leads to structural changes in the latter. Consequently, heterodimerization with any of the three retinoid X receptor (RXR) isoforms occurs, and the newly formed trivalent complex, vitamin D-VDR-RXR, will be subsequently translocated towards the nucleus of the target cell, where it will activate or inhibit gene transcription (5). The VDR gene can regulate up to 200 genes and modulates most effects of vitamin D throughout the body (6). The active form of vitamin D, 1,25-dihydroxyvitamin D (1,25(OH)_2_D) has regulatory immune effects, as it tampers the production of pro-inflammatory cytokines (interleukin (IL)-12, IL-6, IL-17, IL-8, IL-9, tumor necrosis factor-α (TNF-α) and stimulates the release of anti-inflammatory cytokines (including IL-10, IL-4 and IL-5) and the monocyte to macrophage differentiation (1, 7, 8). The infection defense role of 1,25(OH)_2_D is also exerted through T helper (TH) cell 2 production stimulation, activation of antimicrobial peptides (AMPs) and toll-like receptors (TLRs) (9–11). Clinical findings sustain the protective role of 1,25(OH)_2_D against infections, as low concentrations of 25(OH)D have been linked to infections of the respiratory tract, digestive tract or urinary tract and vitamin D supplementation seems to reduce their incidence (12–14). VDR also modulates the effect of T helper cells, as the VDR-RXR complex is recruited by the vitamin D response elements (VDREs). Transcription of messenger ribonucleic acid (mRNA) takes place, which encodes various proteins. The subsequent synthesis of cytokines, cathelicidins and defensins (particular chemoattractants for T cells) will produce a shift from the pro-inflammatory response mediated by TH1 cells towards an anti-inflammatory response modulated by the T regulatory (Treg) cells and by the TH2 cells (15–17). The acknowledgement of these molecular mechanisms has created the premises for the evaluation of VDR gene role in the development of infectious diseases. In particular, children are a population segment which is particularly susceptible to infections at young ages, but their incidence seems to be dependent on genetic factors influencing the individual immune response. Previous studies have shown a potential link between VDR gene polymorphisms and viral respiratory tract infections in children (18, 19). A schematic representation of VDR’s involvement in immune response and the role of VDR’s common polymorphisms in achieving these functions has been provided through **Figure 1**.

Initially, there was little data known regarding the function of VDR gene polymorphisms, and most of them were believed to represent anonymous restriction fragment length polymorphisms (RFLP) (20). The 3’ untranslated region (UTR) of the VDR gene represents a source of multiple polymorphisms, with a variation in their number according to different authors (21, 22). Significant attention has been given to the role of VDR genotypes upon circulating vitamin D levels. The *ApaI* polymorphism (SNP rs7975232) implies a moderate or high risk for vitamin D deficiency, depending on the genotype (23). The *BsmI* polymorphism (SNP rs1544410) regulates the messenger RNA stability. Carriers of the B allele will have a reduced VDR gene expression, which does not necessarily alter vitamin D levels, but makes these individuals less susceptible to vitamin D supplementation, according to a Brazilian study (24). These findings are in line with a pediatric study conducted on children with cystic fibrosis, in whom supplementation with a vitamin D megadose showed benefits over markers of inflammation and oxidative stress, and 25 (OH) vitamin D (25(OH)D) levels. The same study showed how serum 25(OH)D and calcium levels were only improved in BB and Bb genotype carriers of the VDR gene. No differences were seen in serum vitamin D levels, regardless of VDR *BsmI* genotype (3). The *FokI* polymorphism modifies the length and activity of the VDR protein, as it can modify the translation start site (25, 26). In Asian populations, the G/G genotype of the *TaqI* polymorphism was associated with an increased risk of vitamin D insufficiency, but not deficiency and predisposition to injury in athletes (27).

The 25(OH)D levels seem to be influenced by other receptors linked to vitamin D assimilation, such as the vitamin D-binding protein (DBP). DBP represents the major transporter of 25(OH)D, as it carries its majority (85-90%) from the liver to tissues and organs where it accomplishes its functions. There are two well-studied polymorphisms of DBP known, namely rs4588 and rs7041 (28). In a study enrolling a cohort from Saudi Arabia, major homozygous carriers of the rs7041 of DBP showed baseline higher 25(OH)D levels. Individuals with major homozygous genotypes of either rs7041 or rs4588 presented a better response to 25(OH)D supplementation (29). This rs7041 polymorphism has also been associated with severity of COVID-19 infections in children. The GG genotype of the rs7041 polymorphism was mostly predictive of mild disease forms, as opposed to the TT or TG genotypes, which presented higher frequency among the subjects with moderate or severe COVID-19 infections (30).

This narrative review aims to highlight the importance of VDR gene polymorphisms in the susceptibility towards pediatric infections and their related complications, in light of available literature data.

## 2 Methods

A thorough search of PubMed, Web of Science and Google Scholar database was conducted using initially the following key words: “VDR” AND “child” AND “infection”. In order to increase the specificity of the search, given the heterogeneity of the infectious processes studied, the word “infection” was later replaced in each individual search with “respiratory tract infection” OR “pneumonia” OR “bronchiolitis” or “gastroenteritis” OR “diarrhea” OR “urinary tract infection” OR “sepsis”. The articles selected encompassed original research, population-based articles (case-control, longitudinal studies and randomized controlled trials) published in extenso until January 31^st^, 2026, which included a pediatric population and which analyzed the VDR gene polymorphisms in relation to infectious processes and their complications. Only articles published in the English language were included in this review. Rayyan app was used to triage, remove duplicates and select the relevant articles. The current narrative review focused on extracting information regarding the relationship between VDR gene polymorphisms and prevalence of pediatric infections, as well as its connection to vitamin D status and vitamin D supplementation. Vitamin D status was defined as the serum level of 25(OH)D. Studies analyzing only the impact or role of vitamin D status in the incidence and/or recurrence of pediatric infections were excluded, as well as research limited to adult populations or experimental studies. Moreover, the following article types were left out of the result section: review articles, meta-analyses, case reports, case series and correspondence-based articles.

## 3 Results

A total of 21 studies were identified which included study groups of pediatric ages and which met the inclusion and exclusion criteria. These have been synthesized through **Tables 1**–**3**.

**Table 1:** Studies analyzing VDR SNPs in viral/bacterial respiratory tract infections in children without confirmed etiology.

Reference, year	Country	Study design	Pediatric age group	Study groups	SNPs analyzed	Conclusions
Subanada et al., 2020 (19)	Indonesia	Case-control	0–5 years	35 children with ALRIs (bronchiolitis and pneumonia) 35 healthy controls	VDR gene: • *FokI* (rs2228570)	• no significant associations between the *FokI* polymorphism and the risk of ALRIs
Zacharioudaki et al., 2021 (31)	Greece	Prospective, case-control	0–24 months	52 children with viral infection (including 34 children with acute bronchiolitis) 40 children with bacterial infection 40 healthy controls	VDR gene: *FokI* (rs2228570) *TaqI* (rs731236) *BsmI* (rs1544410)	• *TaqI* polymorphism- more frequent in subjects with viral infection when compared to controls
Vorobeva et al., 2025 (35)	Russia	Longitudinal	0–6 months	• children with ALRIs	VDR gene: *FokI* (rs2228570) *TaqI* (rs731236)	• minor TT alleles of the *FokI* gene- high frequency of respiratory tract infections (more than 4 episodes in the first 6 months of life)
Alvarez et al., 2017 (36)	Brazil	Prospective, case-control	0–24 months	181 children with severe bronchiolitis • 536 healthy controls (aged 19–25 years)	•VDR gene: • *FokI* (rs2228570)	• the *FokI* polymorphism was associated with severe bronchiolitis
Jolliffe et al., 2018 (37)	United Kingdom	Longitudinal	0–11 years	725 adults (aged 16–94 years) with RTIs 737 children with RTIs	VDR gene: rs4334089 rs11568820 rs7970314	• the rs4334089 polymorphism- associated with RTI recurrence
Awasthi et al., 2021 (38)	India	Case-control	2–59 months	160 children with CAP 160 healthy controls	VDR gene: *ApaI* (rs7975232) *FokI* (rs2228570) *TaqI* (rs731236) *BsmI* (rs1544410)	• the *FokI* polymorphism was associated with the occurrence of CAP
Abouzeid et al., 2018 (39)	Egypt	Case-control	6 months- 6 years	300 children with CAP 300 healthy controls	VDR gene: • *FokI* (rs2228570)	• the *FokI* polymorphism increases susceptibility for CAP • the *FokI* polymorphism is associated with severe CAP, acute respiratory failure, likelihood of ICU admission and hospital mortality
Roth et al., 2008 (40)	Canada	Case-control	1–24 months	56 children with ALRIs (bronchiolitis and pneumonia) 64 healthy controls	VDR gene: *FokI* (rs2228570) *TaqI* (rs731236)	• both *FokI* and *TaqI* polymorphism increase susceptibility for ALRIs

ALRI, acute lower respiratory tract infections; CAP, community-acquired pneumonia; ICU, intensive care unit; SNP, single nucleotide polymorphism; VDR, vitamin D receptor.

**Table 2:** Studies analyzing VDR SNPs in COVID-19 and RSV infections in children.

Reference, year	Country	Study design	Pediatric age group	Study groups	SNPs analyzed	Conclusions
Chen et al., 2025 (18)	Taiwan	Case-control	0–17 years	123 children with RT-PCR confirmed COVID-19 infection Age-matched healthy controls, unexposed to COVID-19	VDR gene: *ApaI* (rs7975232) *FokI* (rs2228570) *TaqI* (rs731236) *BsmI* (rs1544410)	• FokI polymorphism was related to the PASC score
Giatraki et al., 2025 (30)	Greece	Case-control	1 month-14 years	60 children with laboratory-confirmed COVID-19 infection 60 healthy controls	• VDR gene: *ApaI* (rs7975232) *FokI* (rs2228570) *TaqI* (rs731236) *BsmI* (rs1544410) VDBP: Gc gene rs7041 Gc gene rs4588 *CYP27B1–1260* promoter polymorphism rs10877012	significantly higher prevalence of the *FokI* FF genotype among the COVID-19 group *TaqI* TT genotype, *Gc1F* haplotype- more frequent in the control group the *FokI* FF genotype-correlated with COVID-19 severity
Mohamed et al., 2025 (41)	Egypt	Case-control	0–17 years	77 children with RT-PCR confirmed COVID-19 infection 107 healthy controls	VDR gene: • *FokI* (rs2228570)	• *FokI* TT genotype- correlated with higher COVID-19 incidence and disease severity
Antipkin et al., 2022 (42)	Ukraine	Pilot, open-label	9–16 years	12 children with asymptomatic COVID-19 infection 24 children with mild/moderate COVID-19 infection	VDR gene: *FokI* (rs2228570)	the symptomatic COVID-19 group presented lower vitamin D levels than the asymptomatic group the *FokI* G allele polymorphism seems to positively influence vitamin D supplementation
Kresfelder et al., 2011 (45)	South Africa	Case-control	0–2 years	296 children hospitalized for RSV infection 113 healthy children	VDR gene: *FokI* (rs2228570)	• T allele of *FokI* was more prevalent in the study group • C allele of *FokI* was more prevalent in the control group
Janssen et al., 2007 (46)	Netherlands	Case-control	<1 year of age (most of the study group), 10 children> 1 year	470 children hospitalized for RSV bronchiolitis and their parents (459 mothers, 448 fathers) 1008 healthy controls	VDR gene: *FokI* (rs2228570)	• T allele of *FokI* was associated with severe RSV bronchiolitis

PASC, post-acute sequelae of SARS-CoV-2 infection; RSV, respiratory syncytial virus; RT-PCR, reverse-transcriptase polymerase chain reaction; VDR, vitamin D receptor.

**Table 3:** Studies analyzing VDR SNPs in non-respiratory tract infections in children.

Reference, year	Country	Study design	Pediatric age group	Study groups	SNPs analyzed	Conclusions
Barut et al., 2023 (57)	Turkey	Case-control	Premature born infants	• 74 infants with NEC 147 infants without NEC	VDR gene: *FokI* (rs2228570)	• vitamin D levels were significantly lower in the NEC group TT genotype of the *FokI* polymorphism prevailed in the NEC group
Guzeeva et al., 2019 (61)	Russia	Case-control	12–15 years	19 children with chronic gastroduodenitis and *H. pylori* infection 32 children with chronic gastroduodenitis and without *H. pylori* infection	VDR gene: *ApaI* (rs7975232) *TaqI* (rs731236) • *BsmI* (rs1544410)	• allele B of *BsmI* and allele T of the *TaqI* gene were more frequent among children without *H. pylori*; both alleles were correlated with higher bone mineral density
Aslan et al., 2011 (69)	Turkey	Case-control	0–18 years	92 children diagnosed with UTI 105 children without a history of UTI	• VDR gene: *ApaI* (rs7975232) *FokI* (rs2228570) *TaqI* (rs731236) • *BsmI* (rs1544410)	the f allele of the *FokI* polymorphism is a risk factor for UTI the “a” allele of the *ApaI* polymorphism is a protective factor for UTI
Mahyar et al., 2018 (70)	Iran	Case-control	1 month-12 years	• 60 children with UTI: ✓ 25 children with acute pyelonephritis ✓ 35 children with cystitis • 60 healthy controls	VDR gene: *ApaI* (rs7975232) *FokI* (rs2228570) *TaqI* (rs731236) • *BsmI* (rs1544410)	the b allele of the *BsmI* polymorphism and the A allele of the *ApaI* polymorphism were significantly more frequent in the UTI group the following alleles were significantly more frequent in the acute pyelonephritis group, as opposed to the cystitis group: T allele of *TaqI*, b allele of *BsmI* and a allele of *ApaI*
Shaheen et al., 2022 (71)	Egypt	Case-control	1month-13 years	50 children with sepsis admitted in the PICU 100 healthy controls	VDR gene: *ApaI* (rs7975232) *FokI* (rs2228570) *TaqI* (rs731236) • *BsmI* (rs1544410)	• no significant differences in genotype distribution between the case and control group for neither of the four studied polymorphisms
He et al., 2021 (72)	China	Case-control	0–12 years	267 children with sepsis 283 healthy controls	VDR gene: rs9729 rs2107301 rs2189480 rs2239185 rs3782905 rs4516035 rs7139166 rs11168266 rs11168293	• C alleles of the VDR rs2189480 and rs2107301- protective against sepsis
Tayel et al., 2018 (73)	Egypt	Case-control	Neonates	Study group-80 subjects: 40 neonates with sepsis and their mothers Control group-80 subjects: 40 healthy neonates and their mothers	VDR gene: *FokI* (rs2228570) *TaqI* (rs731236)	T/T genotype of *FokI* was associated with lower vitamin D levels and was linked to sepsis *TaqI* polymorphism did not seem to influence vitamin D levels

*H. pylori, Helicobater pylori*; NEC, necrotizing enterocolitis; PICU, pediatric intensive care unit; UTI, urinary tract infection; VDR, vitamin D receptor.

### 3.1 VDR gene polymorphisms and respiratory tract infections

#### 3.1.1 VDR gene polymorphisms and community acquired respiratory tract infections

There is proof that VDR gene polymorphisms present different genotype distribution in children with bacterial and viral infections. Within a study of Zacharioudaki et al., enrolling infants with bacterial, viral (predominantly involving the respiratory and gastrointestinal tract) and healthy controls, the *TaqI* T allele was significantly more frequent in the viral infection group as opposed to healthy controls. No differences were found between either study group for the *FokI, BsmI*, nor *ApaI* polymorphisms of VDR (common VDR SNPs). Group- specific component 1 fast (Gc1F), an electrophoretic variant of VDBP, had a significantly higher prevalence in the control group when compared to infants with viral infections. No significant differences in genotype frequency were found for VDR and VDBP polymorphisms when comparing the bacterial infection group with controls (31). Still, this study only conducted a comparative assessment of bacterial and viral infections, and did not deliver any information regarding the differential expression of VDR polymorphisms in relation to the location of the infectious process. Its results confirm previous hypothesis suggesting that the Gc1F variant of VDBP is associated with a higher affinity for vitamin D and, consequently, with a normal-ranged vitamin D level (32, 33). Furthermore, *in vitro* studies have showed that vitamin D poses an antiviral effect within the bronchial epithelial cells and that vitamin D supplementation can aid in the treatment and prevention of respiratory tract infections (34). This explains why most of the pediatric research analyzing the relationship between VDR polymorphisms and infectious processes has so far focused on respiratory tract infections, with an emphasis on either one or multiple VDR well-known polymorphisms [*BsmI* (rs1544410), *FokI* (rs2228570, previously known as rs10735810) and *TaqI* (rs731236)]. In a cohort of young Russian infants, aged under 6 months, the *FokI* gene carriers of minor TT alleles were more susceptible towards developing respiratory tract infections, with a frequency of more than 4 episodes during the first half of year of life (35). The *FokI* (rs2228570) polymorphism was also associated with increased risk of severe, even life-threatening bronchiolitis, in research conducted by Alvarez et al. However, as the control group was not age-matched, consisting of healthy adults, the statistical power of the study is relatively limited, considering that bronchiolitis is a condition found at very young ages and that the control group’s history of lower respiratory tract infections was not taken into account (36). An extensive study conducted in the United Kingdom, prenatally enrolled a cohort of 737 children and followed up the participants up to 11 years for their incidence of upper respiratory tract infections, as reported by their primary care physicians. The study researched the distribution of three VDR polymorphisms, namely rs4334089, rs11568820 and rs7970314. The rs4334089 genotype was the only one associated with increased risk of recurrence of upper respiratory tract infections. Still, as the study took into consideration only primary care visits, it is highly unlikely that the real incidence of the infections was captured, as the infectious episodes with more bothersome symptoms were most likely the ones which were supervised and managed by the primary care physicians (37).

Within an Indian pediatric research, enrolling subjects aged between 2 months and 5 years, the dominant CT and CC genotypes of *FokI* (rs2228570) were regarded as risk factors for community acquired pneumonia (CAP). The other VDR gene polymorphisms analyzed, *BsmI* (rs1544410), *TaqI* (rs731236) and *ApaI* (rs7975232) did not predispose towards the development of CAP (38). These findings were in line with another pediatric, Egyptian multicentric study, which showed how the *FokI* (rs2228570) polymorphism modifies susceptibility for CAP development, especially for carriers of the FF genotype, which also presented lower serum vitamin D levels (39). These studies emphasize the predisposing role of the *FokI* (rs2228570) polymorphism in CAP occurrence, for two ethnically different populations (Indian and African Caucasian), but their study groups are limited to subjects who required inpatient care.

Miscellaneous heritage of the enrolled populations leads to different outcomes. In children aged between 1 month and 2 years, the incidence of acute lower respiratory tract infections (especially viral bronchiolitis) was comparatively assessed in terms of *TaqI* (rs731236) and *FokI* (rs2228570) genotypes, compared to a control group without history of such infections. The study, conducted in Canada on a relatively small study sample (70 children), revealed a positive association between the *FokI* ff genotype and the risk of acute lower respiratory tract infections (ALRIs) in infants and toddlers (40). On the other hand, another case-control study conducted in Indonesia, enrolling children with ALRIs (diagnosed with bronchiolitis and pneumonia), younger than 5 years of age, found no significant disparities in the genotype distribution of *FokI*, but acknowledged that low vitamin D and cathelicidin levels constitute risk factors for these infections (19).

The main studies investigating SNPs in the VDR gene associated to respiratory infections in children without confirmed etiologies are summarized in **Table 1**.

Extensive attention has been given regarding the role of VDR gene polymorphisms, their influence on vitamin D receptor and prevalence, as well as the severity of COVID-19 infections. Results of available studies are intriguing, and vary in relation to the age groups studied, the type of and the geographical locations of the studies conducted. In a Greek cohort of children infected with COVID-19, aged between 0 and 14 years, the FokI FF genotype of the VDR was more frequent as opposed to controls. The same genotype correlated with disease severity, whereas the TaqI TT genotype seemed to protect against the infection (30). In contrast, the FokI TT genotype was correlated with higher COVID-19 incidence among a study population of Egyptian children (0–18 years of age), but not with infection severity, in the study of Mohamed et al. (41). Within another study, performed on Taiwanese children (younger than 18 years), the GG genotype of the FokI VDR was associated with less severe pediatric COVID-19 infections (18). In a cohort of school-aged children and teenagers from Ukraine diagnosed with COVID-19 infections, the *FokI* (rs2228570) G allele carriers prevailed, with a prevalence of over 80%. These patients were also regarded as good responders to exogenous vitamin D administration (42). Nevertheless, vitamin D deficiency was predictive of COVID-19 severe forms and adverse outcomes, whereas vitamin D sufficient levels were mostly found in control groups or in children with asymptomatic infections (18, 41–43). These findings are in line with the conclusions drawn by a previous systematic review, which regarded vitamin D levels as predictive of COVID-19 severity and development of multisystem inflammatory syndrome in children (MIS-C) (44). Still, the connection between vitamin D levels and VDR polymorphisms remains questionable. A comparative research conducted on adults with COVID-19 and influenza infections found a direct relationship between lower serum vitamin D levels and the severity of the infections, but concluded that the three polymorphisms of VDR which were studied (rs731236, rs1544410 and rs7975232) did not influence vitamin D serum values (43).

VDR gene polymorphism variation has also been studied in children in relation to the respiratory syncytial virus (RSV). Within a study conducted in South Africa, enrolling children younger than 2 years of age, it was proven that carriers of the T allele of the *FokI* (rs2228570) VDR were predisposed towards developing RSV infection (45). In a study enrolling a similar population from the Netherlands, the same *FokI* (rs2228570) polymorphism was correlated significantly with the incidence of RSV-related bronchiolitis (46). The studies focusing on the impact of VDR gene polymorphisms on COVID-19 and RSV infections have been briefly outlined through **Table 2**.

#### 3.1.2 VDR gene polymorphisms in re-emerging and opportunistic respiratory tract infections

In opportunistic infections such as *Mycobacterium tuberculosis*, the vitamin D-VDR interaction helps in inducing cathelicidin synthesis within the macrophages, which will trigger bacterial annihilation (47). This mechanism can ensure protection against active tuberculosis (TB) infection. Household contacts of patients diagnosed with TB who did not develop the infection were characterized by optimal serum vitamin D levels and increased cathelicidin levels, which declined gradually in 6 months, as the bacterial exposure decreased (48). Therefore, VDR polymorphism can influence the likelihood of active TB infections. Hence, the common VDR SNP polymorphisms were also analyzed in relation to severe and recurrent TB in a population of Indian children. The Tt polymorphism of *TaqI* was considered protective against recurrent TB, whereas none of the polymorphisms analyzed were regarded as risk factors for severe TB (49). Within another case-control Indian study, enrolling individuals aged 11 years or more, the *TaqI* T allele was associated with a positive TB diagnosis, whereas the *FokI* VDR polymorphism was not correlated with a positive diagnosis for this opportunistic infection (50). These findings confirm with the results of another study conducted on a young adult population, in the same geographical region, which failed to identify significant differences in *FokI* genotype and allele variation in subjects diagnosed with multi-drug resistant (MDR) TB in comparison with healthy counterparts (51). Considering that data on the impact of VDR polymorphisms in TB is limited to populations from developing countries, it is difficult to consider these genetic variants as unique risk factors of the infection. Future studies should consider multivariate analysis of multiple, concurring risk factors, including VDR polymorphisms, for a clearer picture of disease vulnerability.

*Bordetella pertussis (B. pertussis)* represents a re-emerging infection, with increased morbidity and mortality at young ages, with an incidence rising in the last decades, principally due to incomplete vaccination coverage (52). In a Dutch study population, enrolling predominantly individuals younger than 14 years of age, the GG genotype of the VDR *Fok*I (rs2228570) polymorphism was substantially more frequent among the study group. The same genotype, as well as the G allele of the *FokI* polymorphism were associated with prolonged symptom duration of at least 4 weeks (53).

### 3.2 VDR polymorphisms in non-respiratory pediatric infections

A summary of studies analyzing the distribution of VDR gene polymorphisms in non-respiratory infections in children has been provided through **Table 3**.

#### 3.2.1 VDR gene polymorphisms and gastrointestinal tract infections

Common VDR SNPs seem to play a role in the prevalence of enteric bacterial infections, through modulation of the intestinal barrier. The role of VDR in the protection against enteric infections was strongly sustained by experimental research conducted on mice. VDR expression yields a protective effect against Salmonella infection of the mouse intestine, through negative modulation of nuclear factor-κB (NF-κB) activity, which is implicated in the pathways responsible for IL-6 secretion (54). Furthermore, both vitamin D and VDR play an important role in maintaining the integrity of the intestinal barrier. In particular, VDR apparently helps in triggering an appropriate immune response of the Paneth intestinal cells to pathogens (55). In vitamin D and VDR deficient mice, inhibition of the Notch pathway aggravated the severity of 2.5% dextran sodium sulfate experimentally induced colitis, through a decrease in claudin-1 and claudin-3 production, two proteins responsible in maintain the tight intestinal inter-epithelial junctions (56). The importance of VDR in preventing intestinal barrier disruption and inflammation has also been evaluated in a study conducted on premature infants with necrotizing enterocolitis (NEC). The TT genotype of the *FokI* VDR polymorphism was associated with a more than four-fold increase in the risk of developing NEC, as compared to a study group of healthy, term-born infants (57).

In human populations, there is a gap of knowledge regarding the impact of common VDR SNPs on the incidence and evolution of viral and bacterial enteritis. In pediatric gastroduodenal pathology, prevalence of vitamin D deficiency is quite high, potentially associated with VDR gene polymorphisms, such as Tt genotype of *TaqI* or the aa genotype of *ApaI*, as suggested by a research conducted on the Ukrainian population (58). Moreover, low serum vitamin D levels have been regarded as risk factors of acute invasive enteritis in children, and adequate vitamin D supplementation might yield protection against bacterial diarrhea (14, 59). Common VDR SNPs, which are involved in the maintenance of the intestinal barrier integrity, as proven through experimental studies, have been linked to inflammatory processes of the intestinal mucosa as well. *TaqI* and *BsmI* polymorphisms were associated with vitamin D levels and inflammatory bowel disease in children, whereas the *FokI* polymorphism correlated with disease activity and severity within the same study (60). In a cohort of children with gastroduodenitis, the relationship between vitamin D levels, VDR gene polymorphisms and the presence of *H. pylori* infection was analyzed. Frequency of the B allele for the VDR *BsmI* polymorphism and t allele of the VDR *TaqI* polymorphism were significantly higher among children without the bacterial infection, and were also associated with higher levels of osteocalcin and bone mineral density (61). Hence, these data support the need for future pediatric research analyzing the role of VDR gene polymorphisms in intestinal inflammations of infectious origin, including gastroenteritis. Carriers of particular SNPs might be more susceptible towards developing more invasive digestive tract infections.

#### 3.2.2 VDR gene polymorphisms and urinary tract infections

Circulating vitamin D aids in the anti-infectious protection of the urothelium, in similar fashion to its antiviral indirect effects, through the production of AMPs (62, 63). These AMPs further confer protection against urinary tract infections (UTIs) through the stimulation of cytokine and β-defensin release (64). The vitamin D-VDR complex also helps in maintaining the integrity of the urothelium through its role in the proper function of the inter-epithelial junctions (65). Previous research has shown that vitamin D deficiency represents a risk factor for first-time and recurrent UTIs in children (66, 67). UTI recurrence can lead to renal scar formation and low serum concentrations of vitamin D could be predictive of renal scarring even from the first febrile UTI episode (68). Therefore, determination of vitamin D levels and genetic factors that modulate its levels could be important from the first UTI episode for anticipation of adverse outcomes. Investigation of the relationship between VDR gene polymorphisms and UTI in children has so far been limited to two studies. Within a case-control study conducted in Turkey, a significantly higher prevalence of the ff *FokI* genotype was identified for the UTI group, whereas the *ApaI* polymorphism was regarded as a protective factor, with the Aa and aa genotypes being more frequent among controls. No significant differences between the two groups were found for the *TaqI* and *BsmI* genotypes (69). Within another study with a similar design, performed in Iran, genotypes of the *ApaI* and *BsmI* polymorphisms were differentially expressed in the case group and also regarded as risk factors for upper UTIs, when a separate comparative analysis was conducted with the lower UTI study sub-group. However, these polymorphisms did not represent risk factors for UTI recurrence and no difference in genotype distribution was seen in relation to renal scarring (70). Both studies had excluded children with congenital anomalies of the kidney and urinary tract (CAKUT), which gives additional relevance to the discrepancies in genotype distribution for the studied pediatric population samples.

#### 3.2.3 VDR gene polymorphisms and sepsis

In terms of severe infections, the role of VDR gene polymorphisms is still incompletely elucidated. One study conducted in Cairo proved that there was no significant variation in genotype frequency for the VDR gene (analysing the common VDR SNPs) between children admitted in the pediatric intensive care units (PICUs) for sepsis and healthy controls. The study completely excluded children with underlying chronic disorders (71). Within another case-control study, conducted on Chinese children, the C alleles of the VDR rs2189480 and rs2107301 were associated with reduced risk of sepsis (72). In case of neonatal sepsis, in an Egyptian study, the VDR *FokI* TT genotype and T allele were regarded as risk factors within the study group. This study also found a correlation between VDR polymorphisms and lower serum vitamin D levels and identified a connection between maternal and neonatal vitamin D deficiency among the study group (73). This study is the only one available in the literature which has investigated a link between VDR polymorphisms and vitamin D levels in pediatric/neonatal sepsis. Previous studies proved how vitamin D deficiency may constitute a risk factor for sepsis, at both neonatal and pediatric ages. In an Indian study, vitamin D levels were inversely related to heart rate, temperature and inflammatory markers such as erythrocyte sedimentation rate (ESR), whereas vitamin D sufficiency was predictive of shorter ventilation support and hospitalization duration (74). In addition, in children with sepsis and vitamin D deficiency the supplementation of a unique dose of 150,000 IU of cholecalciferol can prevent the evolution towards septic shock, according to a randomized controlled trial. The supplement also produced a decrease in IL-6, TNF-α and angiotensin-II (Ang-II) after 8 days, without influencing mortality rates or ventilation duration (75). Still, the available data regarding sepsis and critical illness in children are still inconclusive. Some studies did not find significant differences in VDR gene polymorphisms between children with sepsis and controls, whereas other studies investigating less commonly studied SNPs suggested associations. Moreover, low levels of vitamin D (vitamin D deficiency) have been reported to be associated with worse outcomes or adverse prognosis. In this regard, it is unclear whether VDR gene polymorphisms may be considered independent predictors or if they modulate the effect of low vitamin D levels.

## 4 Limitations of currently available studies and future directions

There are still several knowledge gaps and limitations regarding the association between VDR polymorphisms, vitamin D status and paediatric infections. The studies available in the literature are heterogenous, involving populations from miscellaneous geographical regions, and of different ethnicities and representing various age groups. The small sample sizes for most of the studies limits their reproducibility. The number of subjects included in each study has been subtly highlighted through **Tables 1**–**3**. Hence, future research is required to expand the investigation on larger study populations, with various ethnic backgrounds.

None of the studies included in this review have not performed a multi-variate analysis of concurring risk factors, such as congenital, environmental, socio-economic or nutritional factors which could have also influenced the prevalence of infections. Male gender, history of prematurity, exposure to cigarette smoke and/or to crowded residential environments were more frequent in the study groups for case-control studies conducted on children with lower respiratory tract infections (19, 38, 40). Low maternal education levels and lack of maternal vitamin D supplement intake during pregnancy were identified as risk factors for neonatal sepsis (73). Still, none of these individual factors were assessed in conjunction with VDR polymorphisms, to assess the conjoint risk effects upon infectious disease prevalence.

Most of the research conducted which has analysed the impact of VDR polymorphisms has focused so far on respiratory tract infections, with limited information available regarding viral or bacterial infections of the gastrointestinal tract or urinary tract infections which could display divergent results. In addition, present research has so far failed to deliver picture regarding the relationship between vitamin D status, vitamin D supplementation practices and VDR polymorphisms. Furthermore, longitudinal follow-up of response to the administration of vitamin D supplements in relation to each specific VDR polymorphism could provide additional valuable information. These data may improve the prevention and development of personalized medicine in children and paediatric infectious disease risk stratification.

In recent years, many new studies have assessed the potential impact of VDR polymorphisms in the context of paediatric infections, but a primary focus has been given to the genotype of the *FokI* polymorphism, with fewer studies analysing other polymorphisms of the same gene as well, such as *ApaI, TaqI* or *BsmI*. Thus, future research should be expanded in the direction of other polymorphisms as well.

## 5 Conclusions

Up to date, the most studied VDR polymorphisms in the literature in relation to pediatric infections remain the *BsmI*, *FokI*, *ApaI* and *TaqI* polymorphisms. In particular, most data available is based on studies conducted on respiratory tract infections, especially of viral etiology. A knowledge gap exists regarding the role of VDR in digestive tract infections. Research of the *FokI* polymorphism has so far delivered the most reliable results, as differential prevalence of its SNPs has been found in relation to RSV, COVID-19, UTI and neonatal sepsis incidence. There is insufficient data regarding the link between VDR SNPs and vitamin D status. Hence, it is hard to establish which are the populations at risk of developing vitamin D deficiency, more frequent infections and in whom additional supplementation is required. Moreover, studies have delivered so far contradicting results and are hindered by the heterogeneity of age groups enrolled and largely influenced by the geographical area in which they were conducted. Moreover, multivariate analysis of concurring risk factors is missing from the studies reviewed. Future studies, taking into consideration VDR SNPs, vitamin D status and covariates should provide more insights regarding the utility and applicability of VDR polymorphism detection in pediatric infectious clinical settings.
